# Antibacterial Activity of the Pyrazolone Copper Complex P-FAH-Cu-phen Against *Staphylococcus aureus* and Promotion of Healing of Traumatized Infected Skin in Mice

**DOI:** 10.3390/microorganisms14030659

**Published:** 2026-03-14

**Authors:** Dongyuan Zhou, Changyi Nie, Guancheng Xu, Guoxuan Xie, Marhaba Nurmamat, Tamasha Kurmanjiang, Chunyu Liu, Jinyu Li

**Affiliations:** 1 College of Life Sciences, Xinjiang Normal University, Urumqi 830054, China; zdy19991010@163.com (D.Z.); 18041074466@163.com (C.N.); 17509070211@163.com (G.X.); km260722@163.com (M.N.); tamasha99@163.com (T.K.); m18096838992@163.com (C.L.); 2 Institute of Applied Chemistry, College of Chemistry, Xinjiang University, Urumqi 830046, China; xuguancheng@163.com

**Keywords:** pyrazolone, *Staphylococcus aureus*, virulence factors, biofilm, skin trauma

## Abstract

*Staphylococcus aureus* is a major cause of skin and soft tissue infections, necessitating the development of new topical agents with rapid bactericidal activity and low resistance potential. Here, we evaluated the antibacterial activity of a pyrazolone copper complex (P-FAH-Cu-phen) against *S. aureus*, investigated its in vitro mode of action, and its assessed therapeutic efficacy in a murine model of *S. aureus*-infected skin trauma. P-FAH-Cu-phen exhibited potent bactericidal activity (minimum inhibitory concentration [MIC] 1.4 μg/mL; minimum bactericidal concentration [MBC] 2.8 μg/mL) and rapid killing (>91% eradication within 2.5 min), with no detectable MIC increase under the tested serial passaging conditions. Cell-envelope dysfunction was evidenced by increased supernatant alkaline phosphatase activity, elevated leakage of nucleic acids and proteins, and reduced membrane-associated Na^+^/K^+^- and Ca^2+^/Mg^2+^-ATPase activities. At sub-inhibitory concentrations, P-FAH-Cu-phen reduced haemolytic and coagulase activities, modulated virulence gene expression (*sea*, *hla*, *agrA*), and inhibited biofilm formation and biofilm-associated metabolic activity. In vivo, topical treatment accelerated wound closure and histopathological repair, increased hydroxyproline content, reduced bacterial burden, and lowered TNF-α and IL-10 levels in wound tissues. Collectively, P-FAH-Cu-phen shows multi-faceted anti-infective activity and exhibits further development as a topical candidate for *S. aureus*-infected skin wounds.

## 1. Introduction

*Staphylococcus aureus* (*S. aureus*) is a clinically important pathogen and a common colonizer of human skin and mucosal surfaces [1]. When the integumentary barrier compromises abrasions, burns, surgery, or other trauma, *S. aureus* can readily colonize wound sites and subsequently cause skin and soft tissue infections (SSTIs), often resulting in persistent inflammation, tissue damage, and delayed healing. The pathogenesis of *S. aureus* is multifactorial and encompasses adhesion to and invasion of host tissues, secretion of toxins and enzymes that undermine host defenses, and formation of biofilms [2]. Biofilms increase bacterial tolerance to antibiotics and host immune clearance and consequently are widely implicated in recalcitrant and recurrent infections. Although multiple classes of antibiotics are available clinically, long-term or inappropriate use is associated with adverse effects, the promotion of resistance selection, and reduced patient compliance, which collectively compromise therapeutic outcomes and threaten public health [3,4]. Therefore, there remains a strong need for new antimicrobial candidates with rapid bactericidal activity, low resistance potential, and the ability to attenuate infection-related phenotypes such as virulence and biofilm formation.

Pyrazolones represent a well-studied pharmacophore in medicinal chemistry. Since the introduction of antipyrine as an early antipyretic and analgesic agent, pyrazolone derivatives have garnered sustained interest due to their structural tunability and diverse biological activities [5]. The pyrazolone scaffold is present in a variety of drugs and drug candidates, exhibiting reported antimicrobial and anti-inflammatory properties alongside other pharmacological effects [6]. More recently, pyrazolone-derived Schiff-base ligands have been employed to synthesize metal complexes (e.g., with Cu or Pt), which in some cases exhibit enhanced antibacterial and anticancer activities relative to the parent ligands [7]. However, the manner in which coordination environments and co-ligands shape antibacterial potency and anti-infective phenotypes (including virulence and biofilm-related traits) remains poorly defined, limiting the rational optimization and translational development of these systems.

Previous studies have shown that pyrazolone-based Cu(II) complexes can exhibit antibacterial activity against both Gram-positive and Gram-negative bacteria. For example, Poormohammadi et al. reported measurable antibacterial properties for a series of acylpyrazolone-based Schiff-base copper complexes [8], and we previously reported that the closely related congener P-FAH-Cu-bpy, which shares the same pyrazolone-derived Schiff-base ligand framework but contains 2,2′-bipyridine (bpy) as the co-ligand, also displayed antibacterial activity against *E. coli* and *S. aureus* in separate experimental settings [9]. These findings suggest that certain pyrazolone-based copper complexes can exert bacteriostatic or bactericidal effects, potentially by perturbing essential bacterial processes, although the predominant molecular events and supporting evidence chains often remain unclear across different compounds [10,11]. Introducing copper into such frameworks may further enhance antimicrobial activity, potentially through disruption of metal homeostasis and envelope-associated physiological functions. Accordingly, mechanism-oriented evaluations that link measurable envelope-related readouts, ATPase-related changes, and anti-infective phenotypes are needed to clarify how these complexes act and to guide subsequent structure–activity optimization.

In parallel, metal complexes incorporating aromatic N,N-bidentate chelators such as bpy or 1,10-phenanthroline (phen) have been widely explored as antibacterial agents, and phen-containing complexes have been reported to exhibit antibacterial and antibiofilm activities [12,13,14]. Against this background, the present study focuses on P-FAH-Cu-phen, a Cu(II) complex comprising a pyrazolone Schiff-base ligand (L) and 1,10-phenanthroline (phen) as the co-ligand. Our previous work primarily focused on its antitumor activity [15], while its antibacterial activity against *S. aureus*, anti-virulence effects, biofilm formation inhibition, and therapeutic potential in infected skin wounds have not been systematically evaluated. The previously reported P-FAH-Cu-bpy compound serves as a useful reference within the same structural series; however, because the two compounds were not evaluated side-by-side under identical conditions in the present study, this comparison should be considered qualitative and contextual only.

Here, we assess the antibacterial activity of P-FAH-Cu-phen against *S. aureus* by determining minimum inhibitory concentration (MIC)/minimum bactericidal concentration (MBC) values, time–kill kinetics, and resistance development during serial passaging. We then probe its in vitro mode of action using envelope-associated leakage/enzymatic assays (AKP activity changes, release of nucleic acids and proteins, and Na^+^/K^+^- and Ca^2+^/Mg^2+^-ATPase activities). In addition, we examine anti-virulence and antibiofilm effects at sub-inhibitory concentrations and evaluate topical efficacy in a murine *S. aureus*-infected skin-trauma model by assessing wound closure, bacterial burden, inflammatory markers, and tissue repair–associated indices. Collectively, this work aims to provide an evidence-based foundation for developing pyrazolone-based copper complexes as anti-infective candidates and for informing subsequent design strategies.

## 2. Materials and Methods

### 2.1. Bacterial Strain and Reagent Preparation

*S. aureus* was obtained from the Laboratory of Conservation and Regulatory Biology of Special Environmental Species in Xinjiang (College of Life Sciences, Xinjiang Normal University, Urumqi, China) and cultured on in Luria–Bertani (LB) medium at 37 °C with shaking at 180 rpm for 24 h prior to use.

**Figure 1:**
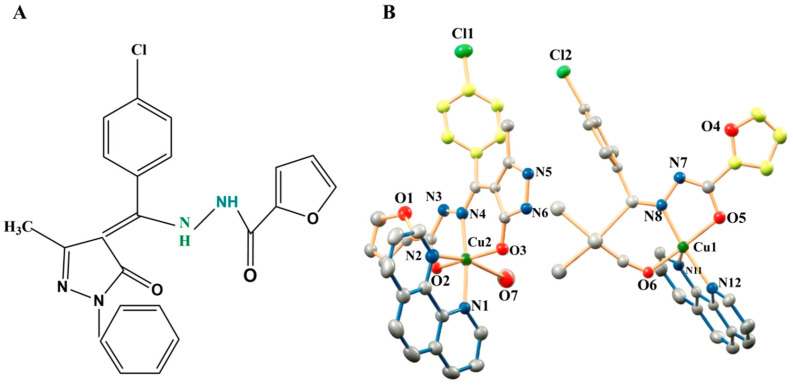
(**A**) Representation of the ligand H_2_L. (**B**) Crystal structure of the P-FAH-Cu-phen complex Novel Copper(II) [15].

Preparation of P-FAH-Cu-phen. P-FAH-Cu-phen was prepared from Cu(OAc)_2_·H_2_O, H_2_L (N-(1-phenyl-3-methyl-4-(4-chlorobenzoyl)-5-pyrazolone)-2-furoic acid hydrazide; Figure 1A), and 1,10-phenanthroline (phen) at a 1:1:1 molar ratio. Cu(OAc)_2_·H_2_O (0.07 mmoL) was dissolved in methanol and added to a MeOH/EtOH (7:3, *v*/*v*) solution (10 mL) containing H_2_L (0.07 mmoL) and phen (0.07 mmoL). The mixture was stirred for 2 h and then left standing at room temperature. Single crystals suitable for X-ray diffraction were obtained by slow evaporation. After approximately 7 days, crystals of [CuL(phen)(CH_3_OH)][CuL(phen)]·CH_3_CH_2_OH·CH_3_OH were collected, washed with cold MeOH/EtOH, and dried under vacuum to constant mass, yielding P-FAH-Cu-phen in approximately 65% yield (Figure 1B). The identity of the complex was consistent with our previous report [15].

Dimethyl sulfoxide (DMSO, analytical grade) was purchased from Tianjin Fuyu Fine Chemical Co. Ltd. (Tianjin, China); Gentamicin was obtained from Yichang Renwei Pharmaceutical Co., Ltd. (Yichang, China); The alkaline phosphatase kit (AKP) and ATPase assay kit (ATPase) assay kit were sourced from Nanjing Jiancheng Bioengineering Institute (Nanjing, China); mupirocin ointment was provided by Hangzhou Zhuyangxin Pharmaceutical Co, Ltd. (Hangzhou, China); barbital sodium (analytical grade) was acquired from Shanghai Reagent No. 3 Factory (Shanghai, China); mouse TNF-α and IL-10 ELISA kits were procured from Jiangsu Meimian Industrial Co., Ltd. (Jiangsu, China); The hydroxyproline assay kit was obtained from Nanjing Jiancheng Bioengineering Institute (Nanjing, China). All other reagents were of analytical grade.

### 2.2. In Vitro Bacterial Inhibition Assay

#### 2.2.1. Agar Diffusion

*Staphylococcus aureus* ATCC 25923 (methicillin-susceptible *S. aureus*, MSSA) was used in this study. The strain was obtained from the Xinjiang Laboratory of Conservation and Regulatory Biology of Special Environmental Species, Xinjiang Normal University. A 1 mL aliquot of frozen stock culture stored at −80 °C was inoculated into 100 mL of LB broth and incubated at 37 °C for 24 h with shaking at 180 rpm for activation. The same strain was used throughout the in vitro assays and the murine wound infection model. The antibacterial activity of P-FAH-Cu-phen was initially assessed using the agar diffusion method according to a previously described procedure with minor modifications [9]. Briefly, a logarithmic-phase bacterial suspension (10^9^ CFU/mL) was mixed with sterile LB medium containing 1.5% agar at a 1:100 (*v*/*v*) ratio and poured into plates. After solidification, sterile filter paper disks (7 mm diameter, Beyotime, Shanghai, China) loaded with 20 μL of P-FAH-Cu-phen solution (200 or 400 μg/mL) were placed on the agar surface and incubated at 37 °C for 24 h. Antibacterial activity was assessed by measuring the diameter of the inhibition zone (DIZ), A disk loaded with 20 μL of 8% (*v*/*v*) DMSO-corresponding to the solvent concentration in the 400 μg/mL P-FAH-Cu-phen solution-served as the control.

#### 2.2.2. Determination of MIC and MBC

The MIC and MBC of P-FAH-Cu-phen were determined using a previously described method with minor modifications [16]. First, the stock solution of P-FAH-Cu-phen (5 mg/mL in DMSO) was diluted with sterile LB medium to 8 μg/mL. Then, serial twofold dilutions were performed to obtain final concentrations ranging from 8 to 0.05 μg/mL. One hundred microliters of each diluted complex solution was then added to 100 μL of the bacterial suspension (adjusted to 2 × 10^6^ CFU/mL). A solvent control containing 0.08% DMSO without the test compound was included to exclude any potential interference from DMSO on bacterial growth. The MIC was defined as the lowest concentration at which no visible bacterial growth was observed after 24 h of incubation at 37 °C, compared to the negative control. Subsequently, 100 μL of the culture from each tube showing no visible growth was transferred to an agar plate for an additional 24 h. The MBC was defined as the lowest concentration of P-FAH-Cu-phen that resulted in no more than five colonies on the agar plate. All tests were performed in triplicate

#### 2.2.3. Plate Experiment

To evaluate the bactericidal effect of P-FAH-Cu-phen after short-term exposure, we performed a short-term viable count assay using a relatively low initial inoculum (1 × 10^4^ CFU/mL) to allow accurate enumeration of surviving colonies, as previously described with minor modifications [17]. Briefly, P-FAH-Cu-phen stock solution (in DMSO) was added to 2 mL of log-phase bacterial suspension to obtain final concentrations of 50, 80, and 100 μg/mL, followed by incubation at 37 °C with shaking (180 rpm) for 3 h. A vehicle control containing 2% (*v*/*v*) DMSO (matched to the 100 μg/mL group) was included. After incubation, 200 μL aliquots were spread onto LB agar and incubated at 37 °C for 24 h. Colonies were then photographed and enumerated. Plate images were analyzed using ImageJ (version 1.54r). Colony numbers were counted manually from plate images using the Cell Counter tool (i.e., no automated threshold-based particle counting was applied). The whole agar plate area was used as the counting region (ROI). Clearly separated colonies were counted as individual colonies, while fused/overlapping colonies were counted conservatively based on visible boundaries. Colonies touching the plate edge were counted only when more than half of the colony area was within the ROI. Colony numbers were then used to calculate bacterial survival according to Formula (1).

(1) $$\mathrm{Bacterial}\;\left(\%\right)=\frac{\mathrm{number}\;\mathrm{of}\;\mathrm{surviving}\;\mathrm{bacteria}}{\mathrm{total}\;\mathrm{number}\;\mathrm{of}\;\mathrm{initial}\;\mathrm{bacteria}}\times 100\%$$

#### 2.2.4. Kinetic Sterilization Curve

The time-kill kinetics of P-FAH-Cu-phen against *S. aureus* were determined using a previously described method [18]. Aliquots (100 µL) of prepared log-phase bacterial suspensions (adjusted to 1 × 10^6^ CFU/mL) were exposed to P-FAH-Cu-phen at concentrations equivalent to 1× MIC or 2× MIC (in triplicate) and incubated at 37 °C for 0.5, 1, 2.5, 5, 10, 20 and 40 min. At each indicated time point, samples were collected by, centrifugation (8000× *g*, 5 min) and washed three times with phosphate-buffered saline (PBS; 0.01 M, pH 7.2). The resulting pellets were resuspended with 100 µL of PBS and subjected to serial 10-fold dilutions in PBS. Aliquots of each dilution were then plated onto LB agar and incubated at 37 °C for 18 h. Colony-forming units (CFUs) were enumerated, and survival rates were calculated using Equation (2).

(2) $$\mathrm{Survival}\;\left(\%\right)=\frac{\mathrm{the}\;\mathrm{number}\;\mathrm{of}\;\mathrm{colonies}\;\mathrm{at}\;\mathrm{time}\;\mathrm{point}}{\mathrm{initial}\;\mathrm{total}\;\mathrm{colonies}}\times 100\%$$

#### 2.2.5. Multi-Step Resistance Screening

To assess the ability of *S. aureus* to develop resistance to P-FAH-Cu-phen after repeated exposure, a multistep resistance selection assay was performed. Using the broth microdilution method [19], logarithmic-phase bacteria were diluted to 10^4^ CFU/mL with sterile LB medium and exposed to sub-MIC levels (0.5× MIC; final DMSO concentration 0.01%, *v*/*v*) of P-FAH-Cu-phen at 37 °C, and the bacteria was continuously cultured for 30 generations. A solvent control containing 0.01% DMSO and a positive control with gentamicin were included. Resistance was defined as a ≥4-fold increase in initial MIC, as previously described.

### 2.3. In Vitro Mode of Action of P-FAH-Cu-phen Against S. aureus

#### 2.3.1. Cell Envelope Disruption Assays (AKP Leakage and Macromolecule Release)

Cell envelope disruption was evaluated by measuring alkaline phosphatase (AKP) leakage and the release of nucleic acids and proteins into the culture supernatant [20]. For AKP leakage, 1 mL of P-FAH-Cu-phen solution at 4× MIC was mixed with 1 mL of logarithmic-phase *S. aureus* suspension (adjusted to 1 × 10^8^ CFU/mL) at a 1:1 (*v*/*v*) ratio to obtain a final concentration of 2× MIC. An untreated suspension mixed with an equal volume of broth served as the control. The mixtures were incubated at 37 °Cwith shaking (180 rpm) for 5 h and then centrifuged (8000× *g*, 5 min) to collect the supernatant. AKP activity in the supernatant was determined using an AKP assay kit (Soleibao, Beijing, China) according to the manufacturer’s instructions, and absorbance was measured at 520 nm using a Multiskan Sky microplate reader (Thermo Scientific, Waltham, MA, USA).

To assess macromolecule leakage, 1 mL of P-FAH-Cu-phen solution at 2× MIC or 4× MIC was added to 1 mL of logarithmic-phase bacterial suspension (1 × 10^8^ CFU/mL) to yield final concentrations of 1× MIC and 2× MIC, respectively, followed by incubation at 37 °C with shaking (180 rpm) for 3 h [20]. After centrifugation (8000× *g*, 10 min), the supernatants were collected. Nucleic acid release was quantified by measuring absorbance at 260 nm using a TU-1900 double-beam spectrophotometer (Purkinje, Beijing, China) [21]. Protein concentration in the supernatant was determined using the Bradford method [22].

#### 2.3.2. Membrane-Associated ATPase Activity Assay

Membrane-associated ATPase activity was assessed using commercial assay kits by measuring enzyme activity in culture supernatants after P-FAH-Cu-phen exposure [20]. Briefly, 1 mL of 4× MIC P-FAH-Cu-phen solution was mixed with 1 mL of logarithmic-phase *S. aureus* suspension (1 × 10^8^ CFU/mL) at a 1:1 (*v*/*v*) ratio to achieve a final concentration of 2× MIC; the untreated mixture served as the control. Samples were incubated at 37 °C with shaking (180 rpm) for 5 h and then centrifuged (8000× *g*, 5 min) to collect the supernatant. Na^+^/K^+^-ATPase and Ca^2+^/Mg^2+^-ATPase activities in the supernatant were determined using an ATPase assay kit (Jiancheng, Nanjing, China) according to the manufacturer’s instructions, and absorbance was measured at 660 nm using a TU-1900 spectrophotometer (Jinghua, Shanghai, China). A 5 h treatment period was used as a common sampling endpoint for the AKP and ATPase-related assays to ensure consistency across these envelope-associated measurements and facilitate direct comparison under the same experimental conditions.

### 2.4. In Vitro Anti-Virulence Properties of P-FAH-Cu-phen Against S. aureus

#### 2.4.1. Effect on Hemolytic Activity of Culture Supernatants of *S. aureus*

The hemolytic activity of *S. aureus* culture supernatants was determined using a previously reported method [23]. Briefly, logarithmic-phase *S. aureus* was treated with different concentrations of P-FAH-Cu-phen (0.25, 0.5, 1, and 2 μg/mL, corresponding to 1/8, 1/4, 1/2, and 1× MIC, respectively) and incubated at 37 °C for 10 min with shaking (130 rpm). After centrifugation at 12,000 rpm for 5 min, the resulting supernatant was filtered through a 0.22 μm sterile membrane filter. Then, 475 μL of the filtered supernatant was transferred to a 1.5 mL centrifuge tube, mixed with 25 μL of sterile rabbit erythrocyte solution, gently vortexed, and incubated at 37 °C for 2 h. Following centrifugation at 3000 rpm for 1 min, 200 μL of the supernatant was carefully transferred to a 96-well plate, and absorbance was measured at 450 nm. The hemolysis rate was calculated using Formula (3). Triton X-100-treated bacterial suspension supernatant served as the positive control, and sterile LB medium processed under the same conditions served as the negative control. The HC_50_ value (concentration required to lyse 50% of erythrocytes) was determined.

(3) $$\mathrm{hemolysis}\;\mathrm{ratio}\;(\%)=\frac{A_{1\;}-\;A_{2}}{A_{3\;}-\;A_{2}}\times 100\%$$

*A*_1_ represents the sample group; *A*_2_ represents the negative control group; *A*_3_ represents the absorbance of the Triton X-100 positive control group.

#### 2.4.2. Effect on Coagulase Activity of *S. aureus*

A drop of 0.5 mL of sterile saline was added to a vial containing lyophilized plasma and shaken until it was completely dissolved. Then, 0.3 mL of *S. aureus* culture solution containing different final concentrations of P-FAH-Cu-phen (0.25, 0.5, 1, and 2 μg/mL, corresponding to 1/8, 1/4, 1/2, and 1× MIC, respectively) was added to the reconstituted plasma and mixed thoroughly. The mixture was incubated at 37 °C, and coagulation was monitored over 6 h, with observations recorded every 30 min. Gentamicin-treated culture served as the positive control, and untreated *S. aureus* culture served as the negative control.

#### 2.4.3. Quantitative Real-Time Polymerase Chain Reaction (qRT-PCR) Analysis of Virulence Factor Expression in *S. aureus*

To analyze the expression of virulence-related genes, logarithmic-phase *S. aureus* was treated with 1× MIC P-FAH-Cu-phen for 30 min or 2 h, with untreated cells serving as the control. Bacterial cells were collected by centrifugation, and total RNA was extracted using a Bacterial Total RNA Extraction Kit (Soleibao Biotechnology Co., Ltd., Beijing, China). cDNA was synthesized using the FastKing RT Kit (with gDNase) (Tiangen Biotech Co., Ltd., Beijing, China). qRT-PCR was performed using SuperReal PreMix Color (SYBR Green) (Tiangen Biotech Co., Ltd., China) to determine the expression of *sea*, *hla*, and *agrA*. Relative gene expression was calculated using the 2^−ΔΔCt^ method, with 16S rRNA acting as the internal reference gene and the untreated group as the calibrator; results were expressed as fold changes relative to the control. Primers were designed by the authors, synthesized by Sangon Biotech (Shanghai) Co., Ltd. (Shanghai, China), and are listed in Table 1. Each qRT-PCR reaction was performed in a 20 μL volume containing 10 μL of 2× SuperReal PreMix Color (SYBR Green), 0.6 μL each of forward and reverse primers (10 μM), 1 μL of cDNA template, and 7.8 μL of RNase-free ddH_2_O. The amplification conditions were as follows: initial denaturation at 95 °C for 15 min; 40 cycles of denaturation at 95 °C for 10 s, annealing at 58 °C for 20 s, and extension at 72 °C for 20 s; and a final extension at 72 °C for 5 min. Melting-curve analysis (60–95 °C) was performed to verify amplicon specificity.

### 2.5. Effects of P-FAH-Cu-phen on S. aureus Biofilm Formation

#### 2.5.1. Crystal Violet Stain

*S. aureus* biofilm formation was determined using a 96-well flat-bottomed polystyrene microtitre plate micromanipulation method [24]. Briefly, different concentrations of P-FAH-Cu-phen (0.125, 0.25, 0.5, 1, 2 μg/mL, corresponding to 1/16, 1/8, 1/4, 1/2, 1 MIC, respectively) were added to 96-well plates containing *S. aureus* suspension (1 × 10^6^ CFU/mL) in a total volume of 200 μL per well and incubated at 37 °C for 24 h. Bacterial cultures without P-FAH-Cu-phen served as the blank control, and gentamicin-treated cultures served as the positive control. After incubation, the optical density at 630 nm (OD_630_) was measured using a microplate reader (Multiskan Thermo Labsystems, Vantaa, Finland) to assess bacterial growth. The supernatant was then carefully removed, and the wells were gently washed three times with PBS. The adhered biofilm was fixed with 99% methanol for 15 min, air-dried, and then stained with 250 μL of 1% (*w*/*v*) crystal violet solution at 37 °C for 5 min. Excess stain was removed by washing three times with ultrapure water, and the plates were air-dried. The bound crystal violet was solubilized by adding 250 μL of 33% (*v*/*v*) acetic acid to each well and incubated for 5 min. Absorbance was measured at 570 nm (OD_570_) using a microplate reader. Biofilm formation was quantified using the biofilm formation index (BFI), calculated according to Formula (4) [25]

(4) $$\mathrm{BFI}=\frac{S\;-\;\mathrm{SC}}{G\;-\;\mathrm{GC}}$$

S: OD_570_ of post-staining sample; SC: OD_570_ of post-staining blank control; G: OD_630_ of pre-staining sample; GC: OD630 of pre-staining blank control.

#### 2.5.2. MTT Assay

The effect of P-FAH-Cu-phen on the metabolic activity of *S. aureus* biofilms was evaluated using the MTT method [26]. Biofilms were established following the same procedure described for the crystal violet assay. Briefly, bacterial suspensions containing different concentrations of P-FAH-Cu-phen (1/16, 1/8, 1/4, 1/2, and 1× MIC) were prepared in 96-well plates and incubated at 37 °C for 24 h. After incubation, the supernatant was carefully removed, and the wells were gently rinsed three times with PBS. Then, 250 μL of MTT solution (0.5 mg/mL in PBS) was added to each well, and the plates were incubated at 37 °C for an additional 3 h. Following the removal of the MTT solution, 250 μL of DMSO was added to each well to dissolve the formazan crystals. Absorbance was measured at 570 nm using a microplate reader. Relative metabolic activity was calculated using Formula (5).

(5) $$\mathrm{Relative}\;\mathrm{metabolic}\;\mathrm{activity}\;(\%)=\frac{{\mathrm{OD}}_{\mathrm{treated}}}{{\mathrm{OD}}_{\mathrm{untreated}}}\times 100\%$$

#### 2.5.3. Bacterial Counting

The inhibitory effect of P-FAH-Cu-phen on *S. aureus* biofilm formation was further assessed using a viable cell counting method [27]. Briefly, bacterial suspensions (total volume 2 mL) containing different concentrations of P-FAH-Cu-phen (1/16, 1/8, 1/4, 1/2, and 1× MIC) were added to 24-well plates and incubated at 37 °C for 24 h. After incubation, the supernatant was carefully removed, and the wells were washed three times with 2 mL of PBS each. For the final wash, 2 mL of sterile PBS was added, and the adherent biofilm was gently scraped off using a sterile cotton swab and thoroughly resuspended. The resulting bacterial suspension was then serially diluted 10-fold in PBS. Aliquots (100 μL) of each dilution were spread onto LB agar plates and incubated at 37 °C for 24 h. CFUs were enumerated, and the biofilm inhibition rate was calculated using Formula (6).

(6) $$\mathrm{Inhibition}\;\mathrm{rate}\;(\%)=\frac{1-\mathrm{number}\;\mathrm{of}\;\mathrm{sample}\;\mathrm{colonies}}{\mathrm{number}\;\mathrm{of}\;\mathrm{blank}\;\mathrm{control}\;\mathrm{colonies}}\times 100\%$$

#### 2.5.4. Scanning Electron Microscopy (SEM, Hitachi, Tokyo, Japan) Observation and Confocal Laser Scanning Microscope (CLSM, Andor, Purbeck, UK)

For biofilm morphology observation, bacterial suspensions containing P-FAH-Cu-phen at 1× MIC and 2× MIC were added to 12-well plates containing sterile glass coverslips; bacterial suspension without P-FAH-Cu-phen served as the blank control. The plates were incubated at 37 °C for 24 h and then gently rinsed three times with PBS.

For SEM, coverslips were fixed in 2.5% glutaraldehyde (prepared in PBS) at 4 °C for 24 h, washed three times with PBS, and then dehydrated through a graded ethanol series (30%, 50%, 70%, 80%, 90%, 95%, and 100%). After freeze-drying, the samples were sputter-coated with gold and observed under a scanning electron microscope to examine biofilm morphology and structure.

For CLSM, coverslips were transferred to new 12-well plates and stained with 1 mL of a mixture of SYTO 9 and propidium iodide (PI) fluorescent dyes. After incubation in the dark for 15 min, the coverslips were observed under a confocal laser scanning microscope to assess bacterial viability and biofilm thickness. The green channel (SYTO 9) was used to detect live bacteria, and the red channel (PI) was used to detect dead bacteria.

### 2.6. Wound-Healing Activity In Vivo

All animal experiments were conducted in accordance with the protocol approved by the Animal Ethics and Welfare Committee of Xinjiang Normal University (Ethic Committee Name: Xinjiang Normal University; Approval Code: XJNU2025LLSC26; Approval Date: 21 August 2025). All procedures were carried out in compliance with the relevant guidelines and regulations for the care and use of laboratory animals, ensuring the welfare of animals throughout the experimental process and minimizing their suffering. Female SPF-grade Kunming mice (*n* = 40, 6–8 weeks old, 18–24 g) were acclimatized for 7 days prior to experimentation. Following anesthesia with 50 mg/kg barbiturates, a full-thickness excisional wound (1.5 cm diameter) was created on the dorsum of each mouse using a previously established method [28] and inoculated with 100 μL of *S. aureus* suspension (1 × 10^9^ CFU/mL) [29].The mice were randomly divided into four groups: an untreated control group (saline), positive control group treated with mupirocin ointment, low concentration group treated with 200 μg/mL P-FAH-Cu-phen, and high concentration group treated with 400 μg/mL Treatments were applied topically daily for 10 days. Wounds were photographed every two days, and wound areas were quantified using ImageJ software (version 1.54r). Images were calibrated using a scale marker, and wound margins were manually delineated using the same boundary definition across all groups and time points (i.e., no threshold-based segmentation was applied). The wound region was defined as the ROI and measured in ImageJ, and wound closure was calculated relative to the initial wound area. (7). At the end of the experiment, mice were euthanized by CO_2_ asphyxiation followed by cervical dislocation. Wound tissues were collected for bacterial enumeration (plate gradient dilution method), inflammatory cytokine measurement (TNF-α and IL-10 ELISA), hydroxyproline content assay, and histological examination (H&E staining).

(7) $$W\mathrm{ound}\;\mathrm{healing}\;(\%)=\frac{W_{0}\;-\;W_{t}}{W_{0}}\times 100\%$$

W_0_ denotes the initial wound area on day 0 and Wt denotes the wound area on day t.

Female SPF-grade Kunming (KM) mice were used in this initial proof-of-concept wound infection study to improve model consistency during longitudinal wound observation and to reduce variability associated with aggressive behavior and wound interference. Mice were randomly assigned to experimental groups. Blinding was not implemented during treatment administration or outcome assessment in the current study. No predefined exclusion criteria were established for this exploratory study, and no animals/data points were excluded from the final analysis.

### 2.7. Statistical Analysis

All experiments were performed in triplicate, and data are presented as mean ± standard deviation (SD). Statistical analyses were performed using SPSS software (version 23.0; IBM, Armonk, NY, USA), and graphs were generated using GraphPad Prism (version 8.0; GraphPad Software, San Diego, CA, USA). Data normality was assessed using the Shapiro–Wilk test. Differences among groups were analyzed by one-way analysis of variance (ANOVA) for normally distributed data, followed by [Tukey’s/Dunnett’s] post hoc test for multiple comparisons. A two-sided *p* < 0.05 was considered statistically significant.

## 3. Results

### 3.1. Antibacterial Activity of P-FAH-Cu-phen

In the agar diffusion assay, no inhibition zone was observed in the DMSO control group, whereas P-FAH-Cu-phen produced inhibition zones of 11.50 ± 0.29 mm at 200 μg/mL and 14.00 ± 0.50 mm at 400 μg/mL.The primary quantitative evaluation of antibacterial activity was based on the MIC and MBC, which were 1.4 μg/mL and 2.8 μg/mL, respectively (MBC/MIC = 2).

**Figure 2:**
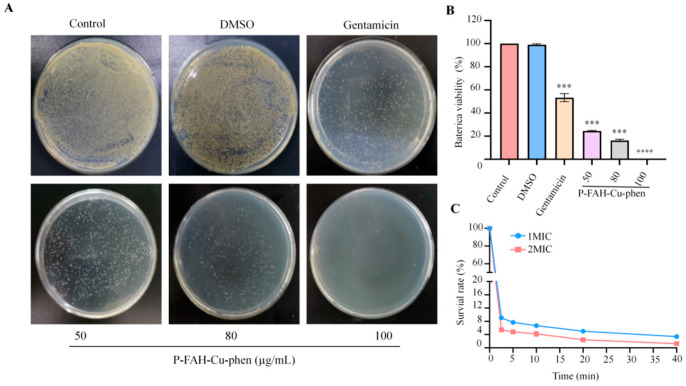
In vitro antibacterial activity of P-FAH-Cu-phen. (**A**) Photographs of *S. aureus* on agar plates. (**B**) Bacteria viability of *S. aureus.* (**C**) Killing kinetics curves of *S. aureus*. *** *p* < 0.001 and **** *p* < 0.0001 when compared to control.

To further evaluate antibacterial activity, colony growth on agar plates was examined after exposure to P-FAH-Cu-phen. Compared with the untreated and DMSO controls, P-FAH-Cu-phen reduced colony formation in a dose-dependent manner (Figure 2A,B), indicating clear growth inhibition of *S. aureus* under the tested conditions. Time-kill analysis further demonstrated rapid bactericidal activity. As shown in Figure 2C, P-FAH-Cu-phen reduced *S. aureus* survival by 91% at 1× MIC and 95% at 2× MIC within 2.5 min, with only 1% of bacteria surviving after 40 min at 2× MIC, demonstrating the rapid bactericidal action of the compound.

### 3.2. Effect of Serial Passaging on the Susceptibility of S. aureus to P-FAH-Cu-phen

**Figure 3:**
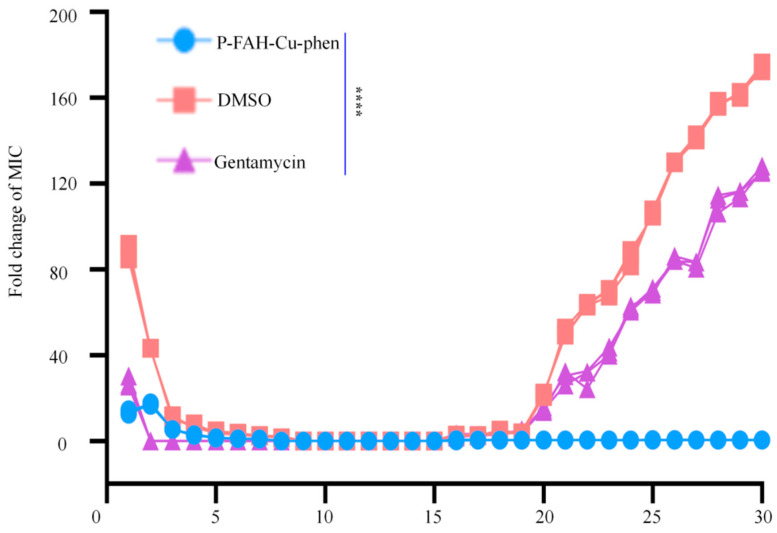
MIC stability of *S. aureus* during serial passaging with P-FAH-Cu-phen. Bacteria were serially passaged for 30 generations in the presence of 0.5× MIC P-FAH-Cu-phen. MICs were measured at regular intervals. DMSO (0.01%) was used as the vehicle control, and gentamicin as a positive control. **** *p* < 0.0001 versus the initial MIC of gentamicin.

To assess adaptive changes during repeated exposure, *S. aureus* was serially passaged in the presence of P-FAH-Cu-phen for 30 generations. As shown in Figure 3, the MIC of P-FAH-Cu-phen fluctuated slightly during the early passages and then remained stable through the later generations, with no detectable overall increase under the tested conditions. In contrast, the MIC of gentamicin increased during serial passaging. DMSO was included as the vehicle control. These data indicate that, under the present serial passaging conditions, repeated exposure to P-FAH-Cu-phen did not produce a measurable MIC shift.

### 3.3. In Vitro Mechanism of Action

#### 3.3.1. Envelope-Associated Dysfunction (AKP Leakage and Macromolecule Release)

**Figure 4:**
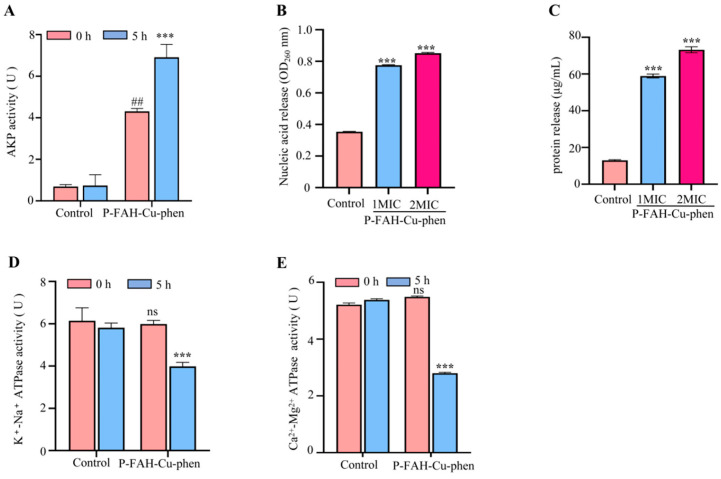
In vitro mechanisms of bacterial inhibition by P-FAH-Cu-phen against *S. aureus*. (**A**) AKP activity after treatment 0 h or 5 h. (**B**) Extracellular nucleic acid (OD_260_ nm) release. (**C**) Extracellular protein content. (**D**) K^+^-Na^+^-ATPase activity after treatment 0 h or 5 h. (**E**) Ca^2+^-Mg^2+^-ATPase activity after treatment 0 h or 5 h. Analysis by two-way ANOVA or one-way ANOVA; *** *p* < 0.001 vs. 5 h group or Control group; ## *p* < 0.01 vs. 0 h group; ns, non-significant.

Alkaline phosphatase (AKP), a cell envelope–associated enzyme, is commonly used as an indicator of increased cell wall/cell envelope permeability, as its extracellular activity rises when the envelope barrier is perturbed [30]. As shown in Figure 4A, exposure to 2× MIC P-FAH-Cu-phen for 5 h increased extracellular AKP activity in *S. aureus* from 4.306 to 6.9065 U/mL prot compared with the untreated control. This increase is consistent with treatment-associated envelope stress accompanied by AKP leakage into the culture supernatant.

Further evidence of envelope-associated dysfunction was provided by the increased extracellular release of nucleic acids and proteins. Relative to the control, nucleic acid release increased by 2.1-fold and 2.4-fold after treatment with 1× MIC and 2× MIC P-FAH-Cu-phen, respectively (Figure 4B). Likewise, the extracellular protein content increased by 4.5-fold and 5.6-fold, respectively (Figure 4C). Collectively, these findings demonstrate that P-FAH-Cu-phen treatment impairs the envelope barrier function of *S. aureus*.

#### 3.3.2. Membrane-Associated ATPase Activity

ATPase-related functions are important for maintaining ion homeostasis and energy-associated physiological processes in bacteria [31,32]. Following 5 h of P-FAH-Cu-phen treatment, the extracellularly measured K^+^-Na^+^-ATPase-related readout in *S. aureus* decreased from 5.9877 to 3.9816 U/mL compared with the untreated control (Figure 4D). Similarly, the Ca^2+^-Mg^2+^-ATPase-related readout declined from 5.3319 to 2.6685 U/mL (Figure 4E). These results indicate that P-FAH-Cu-phen treatment perturbed ATPase-related functions, contributing to envelope-associated dysfunction under the tested conditions.

### 3.4. Anti-Virulence Activity of P-FAH-Cu-phen Against S. aureus

**Figure 5:**
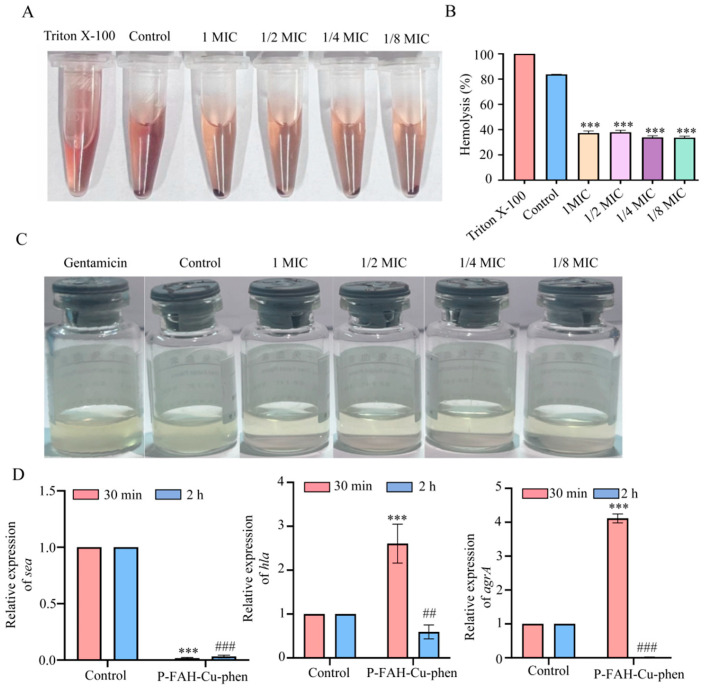
Effects of P-FAH-Cu-phen on Virulence in *S.aureus.* (**A**) Red blood cells hemolysis assay and photographs images for direct ovservation of hemolysis by test samples. (**B**) Hemolysis ratio quantification. (**C**) Coagulase inhibition images. (**D**) qRT-PCR analysis of the expression of virulence factor genes (*sea*, *hla*, and *agrA*) in *S. aureus.* Data are representative of three indepent experiments and analyzed by One-way ANOVA or two ANOVA, *** *p* < 0.001 compared to the Triton X-100 group or the 30 min group. ## *p* < 0.01, ### *p* < 0.001 compared to the 2 h group.

Hemolytic activity of *S. aureus* culture supernatants was assessed to evaluate the effect of P-FAH-Cu-phen on virulence factor production. Hemolysin is a toxin that lyses red blood cells and releases hemoglobin [33]. To determine whether P-FAH-Cu-phen affects hemolytic activity, various concentrations of P-FAH-Cu-phen (0, 1/8, 1/4, 1/2, and 1× MIC) were applied to *S. aureus* for 10 min. The supernatant was then incubated with rabbit erythrocytes for 2 h, and absorbance at 450 nm was measured to calculate hemolysis rates. As shown in Figure 5A, no visible hemolysis was observed in supernatants from P-FAH-Cu-phen-treated cultures, in contrast to the untreated control. Figure 5B shows that P-FAH-Cu-phen significantly reduced hemolytic activity compared with the Triton X-100 positive control, indicating inhibition of hemolysin release. This effect was not strictly dose-dependent under the tested conditions.

Coagulase is an important virulence factor of *S. aureus* that contributes to fibrin clot formation and immune evasion during infection [34]. To assess the impact of P-FAH-Cu-phen on the coagulase activity of *S. aureus*, bacterial cultures containing various concentrations of the compound (1× MIC, 1/2× MIC, 1/4× MIC, and 1/8× MIC) were added to freeze-dried plasma vials. After 30 min of incubation, the vials were gently agitated to evaluate clot formation. As illustrated in Figure 5C, the untreated control group exhibited rapid coagulation, and the gentamicin-treated group also showed coagulation. In contrast, no coagulation was observed in cultures treated with 1× MIC, 1/2× MIC, or 1/4× MIC P-FAH-Cu-phen, while partial coagulation occurred at 1/8× MIC. These findings indicate that P-FAH-Cu-phen treatment was associated with reduced coagulase activity in *S. aureus*, with more pronounced inhibition at higher concentrations.

As illustrated in Figure 5D, qRT-PCR analysis showed that exposure to P-FAH-Cu-phen induced time-dependent changes in the transcription of virulence-related genes (*sea*, *hla*, and *agrA*) in *S. aureus*. Notably, both *hla* and *agrA* exhibited an initial increase in transcript levels at 30 min after treatment. Given the early time point, this response may represent a transient transcriptional adaptation to compound exposure, potentially indicative of an early stress response.

### 3.5. Inhibition of Biofilm Formation by P-FAH-Cu-phen

The effect of P-FAH-Cu-phen on *S. aureus* biofilm formation was evaluated by co-incubating bacteria with the compound for 24 h. This experimental setup assesses the compound’s ability to interfere with biofilm development, rather than its activity against pre-formed mature biofilms.

**Figure 6:**
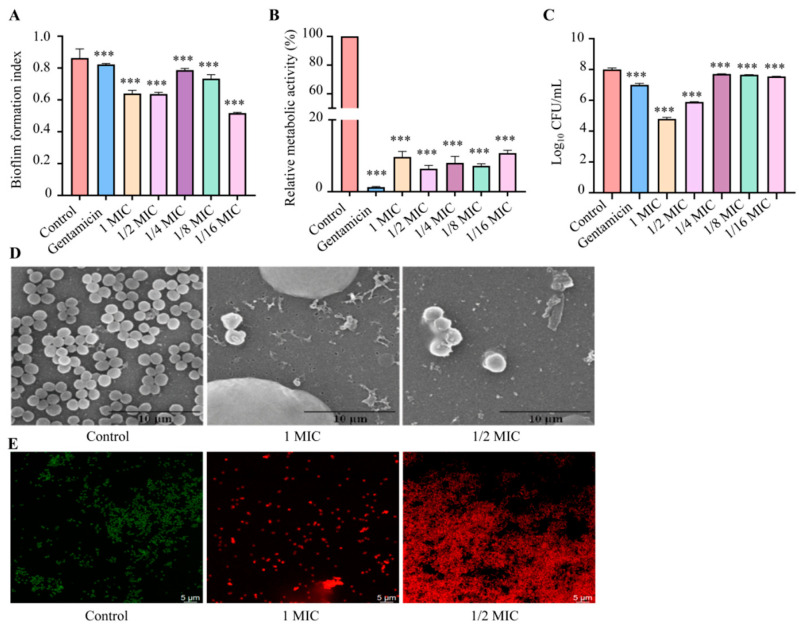
Effect of P-FAH-phen on *S.aureus* biofilms formation. (**A**) Crystal violet staining based quantification of biofilm biomass. (**B**) Quantification of MTT staining. (**C**) Enumeration of viable bacteria in biofilms. (**D**) scanning electron microscope images. (**E**) confocal laser scanning microscope image, *** *p* < 0.001 when compared to Control.

Crystal violet staining showed that P-FAH-Cu-phen reduced biofilm biomass compared with the untreated control, indicating inhibition of biofilm development under the tested conditions (Figure 6A). The MTT assay showed that P-FAH-Cu-phen markedly reduced biofilm-associated metabolic activity after 24 h treatment (Figure 6B), with relative metabolic activities of 9.68%, 6.41%, 8.00%, 7.18%, and 10.76% at 1, 1/2, 1/4, 1/8, and 1/16 MIC, respectively. CFU enumeration demonstrated a significant reduction in the number of cultivable bacteria recovered from biofilms formed under P-FAH-Cu-phen treatment compared to the untreated control (Figure 6C). Taken together, these results indicate that P-FAH-Cu-phen interferes with biofilm formation/development and reduces the metabolic activity and cultivable cell burden of the resulting biofilm-associated population.

SEM supported these findings. In the untreated control, bacteria adhered densely to the coverslip surface, forming multilayered aggregates characteristic of biofilm formation (Figure 6D). In contrast, after treatment with 1× MIC or 1/2× MIC P-FAH-Cu-phen, only sparse bacterial adhesion was observed, with reduced intercellular aggregation and altered cell morphology, indicating impaired surface attachment and microcolony formation. CLSM further confirmed that P-FAH-Cu-phen inhibited biofilm formation: untreated controls showed thick biofilms with abundant live (SYTO9-positive) cells, whereas treated samples exhibited sparse coverage and a predominance of dead (PI-positive) cells (Figure 6E). Collectively, these results demonstrate that P-FAH-Cu-phen effectively inhibits *S. aureus* biofilm formation under the tested co-incubation conditions, reducing biomass, metabolic activity, and viable cell numbers within the developing biofilm.

### 3.6. Experimental Healing of Skin Wounds Infected with S. aureus

**Figure 7:**
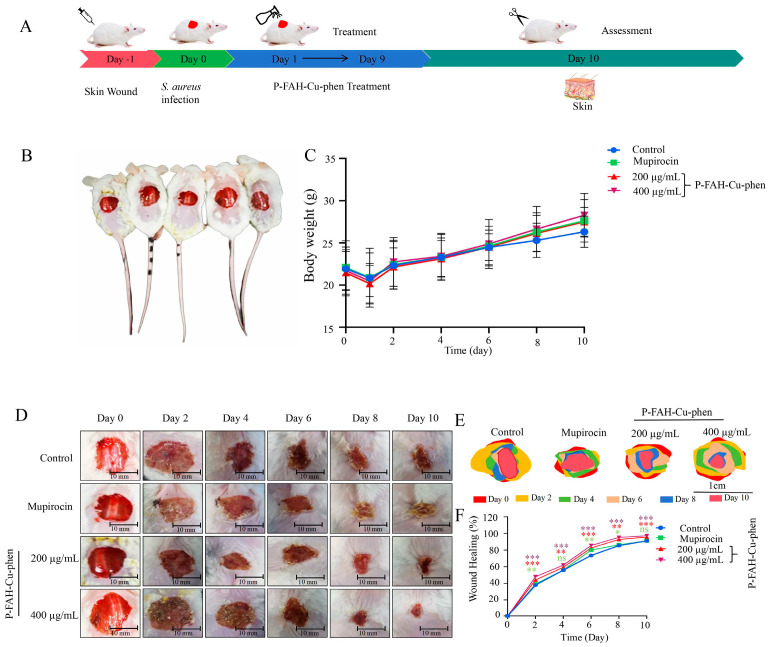
Experimental design and macroscopic wound-healing outcomes of P-FAH-Cu-phen in a murine *S. aureus*-infected wound model. (**A**) Schematic diagram of treatment experiment process for *S. aureus*-infected skin wounds in Mice. (**B**) Mouse skin wound model (**C**) Effect of P-FAH-Cu-phen treatment on body weight of mice. (**D**) Representative photographs of cutaneous abscesses after treatment with P-FAH-Cu-phen or Mupirocin treatment. (**E**) Skin Trauma Schematic. (**F**) The would healing of skin ulcers over 10 days. Data represented as mean ± SD from three independent biological replicates (n = 10). Data are presented as mean ± SD. Statistical significance was analyzed by one-way ANOVA followed by Dunnett’s post hoc test. *p* < 0.05 was considered statistically significant. * *p* < 0.05, ** *p* < 0.01, *** *p* < 0.001, ns, non-significant when compared to Control group.

To evaluate the in vivo therapeutic efficacy of P-FAH-Cu-phen, a full-thickness excisional wound model (1.5 cm diameter) was established on the dorsal skin of KM mice and inoculated with *S. aureus* (Figure 7A,B). Mice were randomly assigned to four groups (n = 10 per group): saline-treated control, mupirocin ointment (positive control), 200 μg/mL P-FAH-Cu-phen, and 400 μg/mL P-FAH-Cu-phen. Wound healing was monitored every two days, and wound areas were quantified from digital photographs using ImageJ software to calculate wound healing rates (Figure 7D–F).

Body weight increased gradually in all groups throughout the treatment period (Figure 7C). Representative wound images showed progressive wound contraction over time (Figure 7D). Quantitative analysis revealed significantly higher wound healing rates in the P-FAH-Cu-phen-treated groups compared with the saline control at the indicated time points, with the 400 μg/mL group exhibiting the fastest wound closure (61.2% on day 4 and 97.8% on day 10) (Figure 7F).

**Figure 8:**
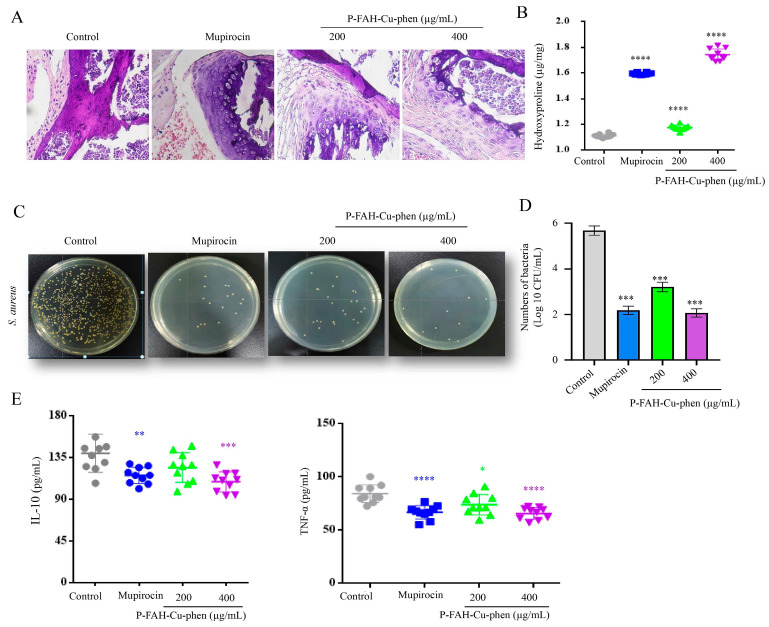
Histological, antibacterial, and inflammatory evaluation of wound tissues after treatment with P-FAH-Cu-phen. (**A**) H&E staining of wound tissue sections from each group at 10 days after the treatment. (**B**) Effect of P-FAH-Cu-phen on hydroxyproline content. (**C**) LB culture pates of different groups of skin after treatment. (**D**) Quantitative analysis of bacteria colony. (**E**) Effects of P-FAH-Cu-phen on the levels of inflammatory factors IL-10 and TNF-α in mouse skin. Data are presented as mean ± SD. Statistical significance was analyzed by one-way ANOVA followed by Dunnett’s post hoc test. *p* < 0.05 was considered statistically significant. * *p* < 0.05, ** *p* < 0.01, *** *p* < 0.001, **** *p* < 0.0001 when compared to Control group.

At the end of treatment (day 10), regenerated skin tissues were collected for histological evaluation and biochemical analyses. H&E staining showed improved re-epithelialization in the mupirocin and P-FAH-Cu-phen groups compared with the saline control, and the 400 μg/mL P-FAH-Cu-phen group displayed more abundant granulation tissue within the wound bed (Figure 8A). Hydroxyproline content in wound tissues was higher in treated groups than in the saline control, with the highest level observed in the 400 μg/mL group (Figure 8B).

Bacterial burden in wound tissue homogenates was assessed by plate counting. Compared with the saline control, colony numbers were markedly reduced in the treatment groups, with the lowest bacterial counts observed in the 400 μg/mL P-FAH-Cu-phen group (Figure 8C,D). Inflammatory cytokines measured in wound tissue homogenates showed that TNF-α and IL-10 levels were lower in the 400 μg/mL P-FAH-Cu-phen group than in the saline control (Figure 8E).

Collectively, P-FAH-Cu-phen treatment was associated with accelerated wound closure and reduced bacterial burden in *S. aureus*-infected skin wounds.

## 4. Discussion

Pyrazolone metal complexes belong to the class of Schiff base compounds, which can coordinate with various metal ions to form complex structures with enhanced biological activity [31]. Research indicates that pyrazolones and their derivatives exhibit a wide range of biological functions, including antibacterial, antifungal, anticancer, antitumor, antiviral, antioxidant, and antituberculosis activities. Moreover, they have the potential to overcome the resistance and toxic side effects associated with traditional antimicrobial drugs, positioning them as promising candidates for novel antimicrobial agents with significant application prospects [35]. Nevertheless, research on the antimicrobial properties of pyrazolone metal complexes remains limited, particularly concerning their mechanisms of action and efficacy against infections on compromised skin.

In the present study, P-FAH-Cu-phen exhibited strong antimicrobial activity against *S. aureus*, with MIC and MBC values of 1.4 and 2.8 μg/mL, respectively. Typically, a bacteriostatic MBC/MIC ratio is ≥4, whereas a bactericidal MBC/MIC ratio is 1 or 2 [36]. The MBC/MIC ratio of 2 determined for P-FAH-Cu-phen confirms its bactericidal activity against *S. aureus*. The bactericidal and inhibitory properties of P-FAH-Cu-phen were further confirmed through both plate assay and time-kill assay (Figure 2). Notably, exposure to the complex for 40 min reduced the bacterial population by 99%, demonstrating rapid bactericidal action compared with conventional antimicrobial agents.

The emergence of bacterial resistance to antimicrobials is a significant factor contributing to the reduced efficacy of antibiotics. Following the assessment of the antimicrobial activity of P-FAH-Cu-phen against *S. aureus*, we examined whether serial passage at sub-MIC levels would alter the susceptibility of *S. aureus* to this compound. Using a multistep passaging assay, no detectable MIC increase was observed for P-FAH-Cu-phen under the tested serial passaging conditions (Figure 3). In contrast, the MIC of gentamicin increased substantially under the same conditions, consistent with the known propensity of *S. aureus* to develop reduced susceptibility to conventional antibiotics [37,38]. However, it is important to note that the current assay evaluates MIC shifts only and does not by itself distinguish stable genetic resistance from tolerance, persistence, or transient adaptive stress responses. Further studies—including assessments of recovery after drug withdrawal, fitness analyses, genomic sequencing, and the phenotypic characterization of passaged isolates—will be necessary to fully define the adaptive potential of *S. aureus* in response to P-FAH-Cu-phen [37,38].

Pyrazolone-based scaffolds are widely represented in bioactive molecules and drug-like compounds, and their aromatic/π-conjugated frameworks together with hydrogen-bonding functionality may facilitate interactions with diverse biomolecular targets [39,40,41,42]. Consistent with this general premise, our mechanistic assays suggest that P-FAH-Cu-phen induces envelope-associated dysfunction in *S. aureus*. Specifically, extracellular AKP activity increased after treatment, which is consistent with envelope stress and leakage of envelope-associated enzymes. In parallel, the marked elevation of nucleic acids and proteins in the culture supernatant further supports leakage-associated changes in envelope barrier function under the tested conditions. In addition, ATPase-related readouts measured in the culture supernatant (K^+^-Na^+^-ATPase and Ca^2+^-Mg^2+^-ATPase) decreased after exposure to P-FAH-Cu-phen, suggesting perturbation of ion-homeostasis- and energy-related processes [43,44]. Our mechanistic assays suggest envelope-associated dysfunction in *S. aureus*, supported by increased AKP leakage, macromolecule release, higher conductivity, and altered ATPase-related readouts in supernatants. Because these measures are indirect and ATPase readouts were not obtained from isolated membrane fractions, the data do not establish primary membrane targeting; direct membrane integrity assays are needed to clarify the primary target.

Notably, we previously reported a closely related congener, P-FAH-Cu-bpy, in which 1,10-phenanthroline is replaced by 2, 2′-bipyridine. Despite this co-ligand substitution, P-FAH-Cu-bpy exhibited comparably potent antibacterial activity and rapid killing, together with a similar set of mechanistic signatures, including increased leakage markers (e.g., AKP activity and release of nucleic acids/proteins), decreased ATPase activities, elevated intracellular Cu accumulation, and a low propensity for resistance development upon serial passaging [9]. Taken together, these cross-study consistencies support a scaffold-class effect dominated by the shared Cu-pyrazolone framework, while the co-ligand (phen vs. bpy) likely tunes physicochemical properties (e.g., stability/lipophilicity and uptake) rather than fundamentally changing the qualitative antibacterial phenotype.

*S. aureus* produces multiple virulence factors, including hemolysins, enterotoxins, and coagulase, which contribute to host damage and persistence [45]. α-Hemolysin is a key mediator of cytotoxicity, and strategies that reduce hemolysis may attenuate pathogenicity independently of growth inhibition [46,47]. In this study, P-FAH-Cu-phen reduced hemolytic activity in *S. aureus* culture supernatants, including at sub-MIC, indicating suppression of a toxin-associated phenotype under conditions where bactericidal effects are minimal. P-FAH-Cu-phen also reduced coagulase activity, although this effect was less pronounced at sub-MIC levels, suggesting that reduced bacterial fitness at higher concentrations may contribute to the observed phenotype [48]. qRT-PCR showed time-dependent changes in *sea, hla,* and *agrA* (a key regulator in the agr quorum-sensing system) [49]. At 30 min, hla and agrA were transiently upregulated, which may reflect an early stress response or adaptive feedback. By 2 h, the expression of all three genes was downregulated, including reduced expression of the toxin-associated genes sea and hla [50]. These transcriptional changes were observed at 1× MIC, a concentration at which bacterial fitness and global transcription may be affected. Under these bactericidal conditions, the observed modulation of virulence-related gene expression likely reflects a combination of direct compound effects and broader cellular stress responses.

Biofilm formation significantly enhances bacterial tolerance to antibiotics and host immune defenses, contributing to the persistence of chronic infections [51]. In this study, we evaluated the effect of P-FAH-Cu-phen on *S. aureus* biofilm formation during a 24-h incubation using complementary assays, including crystal violet staining, the MTT assay, CFU enumeration, and microscopic imaging. The results demonstrate that P-FAH-Cu-phen effectively inhibits biofilm formation, reducing biomass, metabolic activity, and viable cell counts within the developing biofilm.

Notably, the inhibitory effects were not entirely concordant across different assays: crystal violet staining showed reduced biomass at 1/16× MIC, whereas changes in MTT readouts and CFU counts at this concentration were relatively limited. This partial discrepancy is not unexpected, as these three methods assess different aspects of biofilms-matrix-associated biomass, cellular metabolic activity, and cultivable cell numbers—each of which may be differentially affected by drug treatment due to biofilm heterogeneity. Similar non-linear concentration–response patterns have been reported for small-molecule perturbations in other bacterial systems [52].

It should be emphasized that the present study assessed inhibition of biofilm formation during co-incubation with P-FAH-Cu-phen, rather than the eradication of pre-established mature biofilms. Mature biofilms are clinically more relevant in chronic infections; therefore, while our findings suggest that P-FAH-Cu-phen has potential to prevent biofilm development, its efficacy against established biofilms requires further investigation. The non-monotonic concentration responses observed across different assays also reflect the inherent complexity of biofilm development and underscore the value of using multiple complementary methods to evaluate antibiofilm activity.

The integumentary barrier provides a first line of defense against invading pathogens; when disrupted by abrasions, burns, or other injuries, opportunistic bacteria such as *S. aureus* can colonize the wound bed and delay repair. Using an *S. aureus*-infected full-thickness skin trauma model, we found that topical P-FAH-Cu-phen accelerated wound closure and improved histological features of repair, including re-epithelialization and granulation tissue formation, accompanied by increased hydroxyproline content. Because hydroxyproline is a major collagen-associated amino acid, tissue hydroxyproline is widely used as an indirect indicator of collagen deposition during wound healing [53]. In parallel, P-FAH-Cu-phen reduced end-point bacterial burden in wound tissues and was associated with lower local TNF-α and IL-10 levels compared with infected controls. These results are consistent with reduced infection-related inflammatory responses and improved tissue repair [54,55,56]. IL-10 is an important anti-inflammatory cytokine that plays a crucial role in balancing immune responses and preventing excessive inflammation. A reduction in IL-10 could suggest a shift towards a more pro-inflammatory environment or a failure in the anti-inflammatory response. In the context of wound healing, IL-10 reduction may not necessarily correlate with an overall increase in inflammation; rather, it could indicate a disruption in the delicate balance between pro- and anti-inflammatory mediators. Notably, the present data do not distinguish whether the beneficial effects are driven primarily by reduced bacterial load or by altered host responses, and the underlying mechanisms warrant further investigation.

Several limitations of the present study should be acknowledged. First, all experiments were conducted using a single reference strain of *S. aureus*; whether the observed effects extend to clinical isolates, including MRSA, remains to be determined. Second, speciation analysis of the complex in solution was not performed, so the relative contributions of the intact complex versus its dissociated species to the observed activity remain unclear. Third, the in vivo experimental design can be further improved: future studies will include both sexes, blinded outcome assessment, non-infected wound controls, and comprehensive evaluations of local and systemic safety (e.g., skin toxicity, copper exposure, tissue accumulation). Addressing these points in future work will help to more fully establish the therapeutic potential of P-FAH-Cu-phen.

## 5. Conclusions

**Figure 9:**
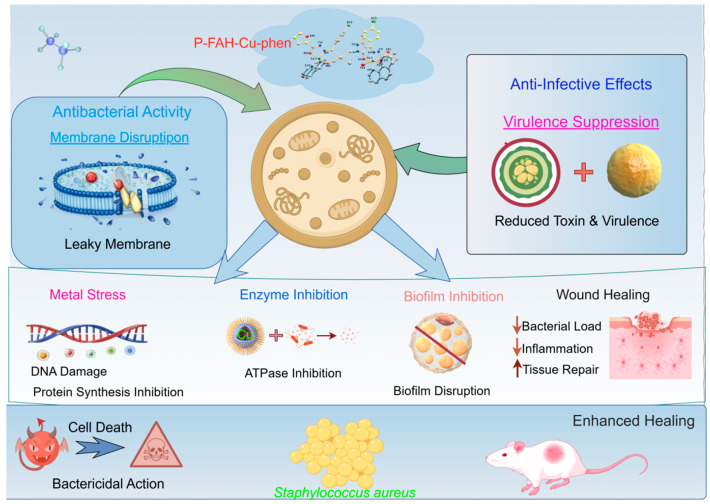
Mechanism Diagram of P-FAH-Cu-phen against *Staphylococcus aureus*.

In summary, P-FAH-Cu-phen exhibited rapid bactericidal activity against *S.aureus* and produced integrated in vitro effects consistent with cell-envelope disruption, impairment of membrane-associated functions, and attenuation of virulence- and biofilm-related phenotypes. In an *S. aureus*-infected skin-trauma model, topical administration accelerated wound closure, improved histological repair, and increased hydroxyproline content, while reducing end-point bacterial burden and infection-associated cytokine levels. Collectively, these findings support further investigation of P-FAH-Cu-phen as a potential topical candidate for *S. aureus*-infected skin wounds (Figure 9).

## Figures and Tables

**Table 1:** qRT-PCR primmers.

Gene Code	Forvard Primers Sequence	Reverse Primers Sequence	Amplicon Size (bp)
*16S rRNA*	GCTGCCCTTTGTATTGTC	AGATGTTGGGTTAAGT CCC	187
*sea*	ATGGTGCTTATTATGGTTATC	CGTTTCCAAAGGTACTGTATT	426
*hla*	TTGGTGCAAATGTTTC	TCACTTTCCAGCCTACT	312
*agrA*	TGATAATCCTTATGAGGTGCTT	CACTGTGCTCGTAACGAAA	259

## Data Availability

The original contributions presented in this study are included in the article. Further inquiries can be directed to the corresponding author.
